# Metabolic Stress Responses in *Drosophila* Are Modulated by Brain Neurosecretory Cells That Produce Multiple Neuropeptides

**DOI:** 10.1371/journal.pone.0011480

**Published:** 2010-07-08

**Authors:** Lily Kahsai, Neval Kapan, Heinrich Dircksen, Åsa M. E. Winther, Dick R. Nässel

**Affiliations:** Department of Zoology, Stockholm University, Stockholm, Sweden; Katholieke Universiteit Leuven, Belgium

## Abstract

In *Drosophila*, neurosecretory cells that release peptide hormones play a prominent role in the regulation of development, growth, metabolism, and reproduction. Several types of peptidergic neurosecretory cells have been identified in the brain of *Drosophila* with release sites in the corpora cardiaca and anterior aorta. We show here that in adult flies the products of three neuropeptide precursors are colocalized in five pairs of large protocerebral neurosecretory cells in two clusters (designated ipc-1 and ipc-2a): *Drosophila* tachykinin (DTK), short neuropeptide F (sNPF) and ion transport peptide (ITP). These peptides were detected by immunocytochemistry in combination with GFP expression driven by the enhancer trap Gal4 lines c929 and Kurs-6, both of which are expressed in ipc-1 and 2a cells. This mix of colocalized peptides with seemingly unrelated functions is intriguing and prompted us to initiate analysis of the function of the ten neurosecretory cells. We investigated the role of peptide signaling from large ipc-1 and 2a cells in stress responses by monitoring the effect of starvation and desiccation in flies with levels of DTK or sNPF diminished by RNA interference. Using the Gal4-UAS system we targeted the peptide knockdown specifically to ipc-1 and 2a cells with the c929 and Kurs-6 drivers. Flies with reduced DTK or sNPF levels in these cells displayed decreased survival time at desiccation and starvation, as well as increased water loss at desiccation. Our data suggest that homeostasis during metabolic stress requires intact peptide signaling by ipc-1 and 2a neurosecretory cells.

## Introduction

Hormonal regulation of development, growth, metabolism and reproduction has been extensively studied in insects, including *Drosophila* (reviewed in [1], [2], [3], [4], [5], [6], [7], [8]). Many types of neurosecretory cells (NSCs) that release peptide hormones into the circulation have been identified in the brain of various insect species [4], [9], [10], [11], [12], [13]. The brain complement of NSCs is complex with cell bodies in several locations and axonal projections to different neurohemal release sites in contact with the circulation. In the larval brain of *Drosophila* the anatomy of NSCs has been comprehensively described from Gal4 enhancer trap lines [9], whereas the anatomy in the adult brain is less clear and based mainly on immunocytochemical mapping of certain neuropeptides (see [14], [15], [16], [17]). More detailed data on adult anatomy of brain NSCs is available from another dipteran insect, the blowfly *Protophormia terraenovae*, based on tracer backfilling from cut nerves [10], [11]. Already the anatomy suggests that in the adult brain NSCs are functionally heterogeneous and efforts so far to map different hormonal peptides to the *Drosophila* NSCs has certainly underscored the complexity of the NSC systems in the brain.

In the adult brain of *Drosophila* a combination of *in situ* hybridization and immunocytochemical mapping and mass spectrometry of dissected corpora cardiaca and corpora allata has revealed a set of peptide hormones derived from NSCs: corazonin, dromyosuppressin (DMS), insulin-like peptides (DILP-2, 3, 5), ion transport peptide (ITP), short neuropeptide F (sNPF) and the peptide products PK-2 of the *hugin* gene and PK-1 of the *Capa* gene [9], [15], [16], [17], [18], [19], [20], [21]. Similar experiments performed for the larval brain NSCs identified the same peptides, as well as the diuretic hormone (DH_44_), sulfakinin (DSK), eclosion hormone, and prothoracicotropic hormone [15], [16], [17], [18], [19], [21], [22], [23], [24], [25]. The extra peptide hormones in larvae are likely to play developmental roles. Additionally there are intrinsic endocrine cells in the corpora cardiaca of both larvae and adults that produce adipokinetic hormone (AKH).

Recent experimental work on non-developmental aspects of brain-derived hormones in *Drosophila* has focused mainly on the roles of AKH in metabolism and DILPs in metabolism, growth, stress resistance and life span [2], [3], [26], [27], [28], [29], [30]. The hormonal peptides known to act on the renal tubules to control secretion in *Drosophila* are mainly released from neurosecretory cells in the ventral nerve cord [22], [31], [32], [33]. Thus, there are several brain peptide hormones whose functions have not been explored.

We demonstrate here that five pairs of large neurosecretory cells in the adult *Drosophila* brain produce colocalized neuropeptides derived from three precursor genes: *dtk*, *snpf* and *itp*. The peptide products from these genes are: *Drosophila* tachykinins (DTKs), short neuropeptide F isoforms (sNPFs) and ion transport peptide (ITP). This is a remarkable combination of peptides considering what is so far known about the disparate functions of these peptides [24], [34], [35].

The DTKs are widely distributed in brain interneurons of insects and crustaceans and have a variety functions as neuromodulators in the visual and olfactory systems as well as in circuits controlling locomotor activity [34], [36], [37], [38], [39]. Until now no brain neurosecretory cells expressing DTKs were found in *Drosophila* [37]. DTKs have also been detected in endocrine cells of the *Drosophila* intestine and display myostimulatory action on the gut muscle [40]. The sNPFs appear to be predominantly expressed in numerous small interneurons in the CNS, subsets of olfactory sensory neurons and in a few brain neurosecretory cells [24]. These peptides are likely to be cotransmitters in circuits of for instance antennal lobe, mushroom bodies, central complex and clock circuits, but have also been implicated in regulation of insulin signaling, feeding and growth [24], [41], [42], [43], [44]. Finally, the *Drosophila* ITPs are located in a small number of brain interneurons and neurosecretory cells and are likely to be antidiuretic hormones that regulate ion transport in hindgut epithelium, and may also have roles in clock circuits [15], [35], [41]. Thus, our discovery here that these seemingly disparate peptides are colocalized in neurosecretory cells prompted us to approach the functional roles of the ten cells producing the peptide cocktail.

The ten large neurosecretory cells are the only ones colocalizing the three peptide gene products that are included in the expression patterns of the two enhancer trap Gal4 lines c929 and Kurs-6 in adult flies. Hence, we could use these Gal4 lines to drive RNA interference (RNAi) specific for *dtk* and *snpf* and monitor the effects of diminishing these peptides in the ten large neurosecretory cells. We asked whether these peptides produce a concerted action in the fly and set out to monitor effects of peptide knock-down on responses to desiccation and starvation. Interestingly, only four pairs of large ITP immunoreactive neurosecretory cells are present in the larval brain, but these do not co-express sNPF, DTKs or c929 at this stage, suggesting that the co-action of the three neuropeptides as possible hormones is a feature specific to the adult fly.

## Results

### Colocalization of ITP, DTK and sNPF in adult brain neurosecretory cells

**Figure 1:**
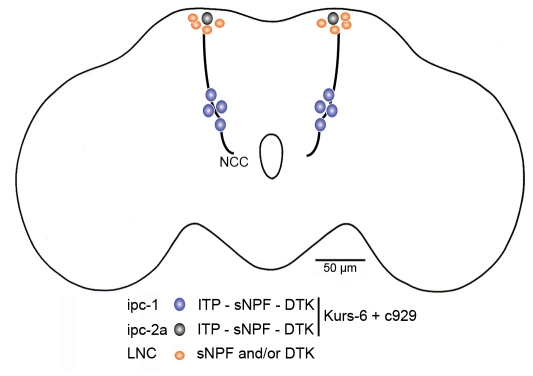
Schematic depiction of a subset of peptidergic lateral neurosecretory cells in the adult *Drosophila* brain. The ipc-1 and ipc-2a cells have large cell bodies and co-express the peptides ITP, DTK and sNPF, as well as the enhancer trap Gal4 lines c929 (transcription factor DIMM) and Kurs-6. These neurosecretory cells have axons extending through the corpora cardiaca nerves (NCC) to varicose terminations in the corpora cardiaca, anterior aorta and anterior intestine. Another set of neurons (LNC) in the same cluster as ipc-2a express either sNPF or DTK, but not ITP, c929 or Kurs-6. Their axon trajectories are not known.

**Figure 2:**
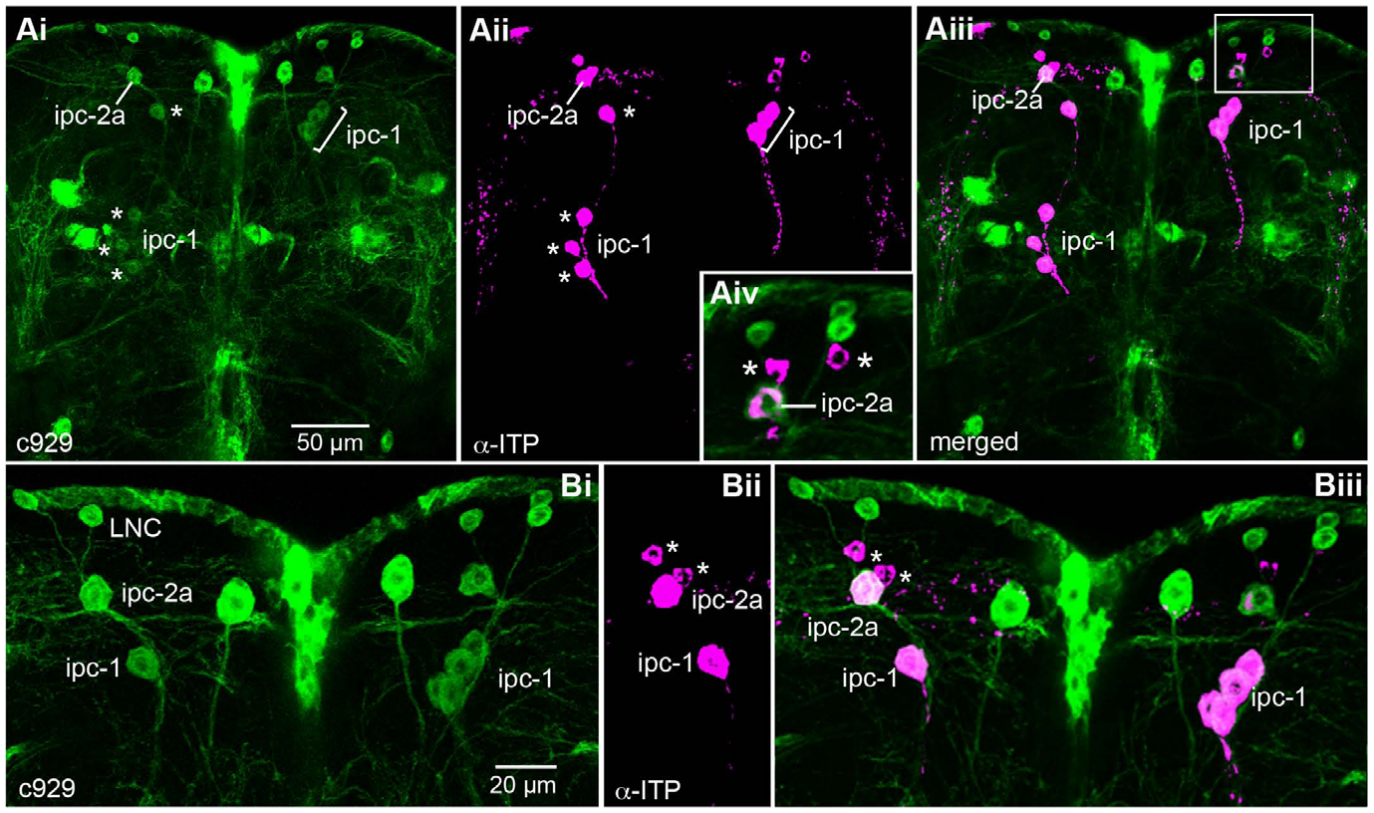
A set of ten large neurosecretory cells coexpress ITP and the transcription factor DIMM. Coexpression of c929-driven GFP (Green; representing transcription factor DIMM) and immunolabeling with antiserum to ITP (magenta) in a subset of neurosecretory cells (ipc-1 and 2a) of the adult *Drosophila* brain. The images are from frontal views of wholemount specimens (dorsal is up). **Ai - iii** Overview of dorsal brain with ipc-1 (asterisks) and 2a neurons with coexpression of ITP and c929 (stack of several confocal sections). Note that cell bodies of the ipc-1 neurons have variable positions (along the same axonal tract; see also Fig. S1). **Aiv** Enlarged view of area that is boxed in Aiii. Note that there are three more ITP-labeled ipc-2 neurons (two seen at asterisks; the third is in adjacent optical section) that do not express c929. **Bi - iii** Higher magnification of the same section with colocalized markers in ipc-1 and 2a cells. Only one ipc-1 is seen to the left in this optical section and four to the right. The asterisks indicate two of the small ipc-2 cells that do not express c929-GFP. A set of presumed lateral neurosecretory cells (LNC) express c929, but not ITP.

A set of large neurosecretory cells were discovered in the brain of *Drosophila* by labeling with antiserum to locust ion transport peptide (ITP) [15]. In the adult brain these are located posteriorly in two clusters of lateral neurosecretory cells (LNCs) and were designated the ITP-immunoreactive protocerebral neurons-1 and 2 (ipc-1 and ipc-2). There are four pairs of ipc-1 neurons and four pairs of ipc-2. The ipc-1 all have large cell bodies (more than 25 µm in diameter), whereas three of the ipc-2 neurons in each group are smaller and one has a cell body of a size similar to the ipc-1 neurons (Fig. 1, 2). Both groups of ipc neurons extend axons to terminations in neurohemal release sites in the corpora cardiaca, corpora allata, aorta and anterior intestine [15].

We used ITP immunocytochemistry in combination with c929-Gal4 directed green fluorescent protein (GFP) to determine whether the ipc-1 and 2a neurons are part of a population of neurons expressing the transcription factor DIMMED (DIMM). It has been shown previously that DIMM expression in *Drosophila* is correlated with a specific peptidergic phenotype: large neurons or neurosecretory cells that produce amidated peptides and are likely to have high secretory activity [45], [46]. The enhancer trap Gal4 line c929 provides a good map of DIMM expressing neurons [45].

As shown in Fig. 2, the four pairs of ipc-1 neurons and one pair of large ipc-2 neurons coexpress c929 driven GFP and ITP-immunoreactivity, suggesting that these cells are DIMM positive. To specify the DIMM expressing large ipc-2 neurons from the smaller ones that are DIMM negative, we designate these neurons ipc-2a.

**Figure 3:**
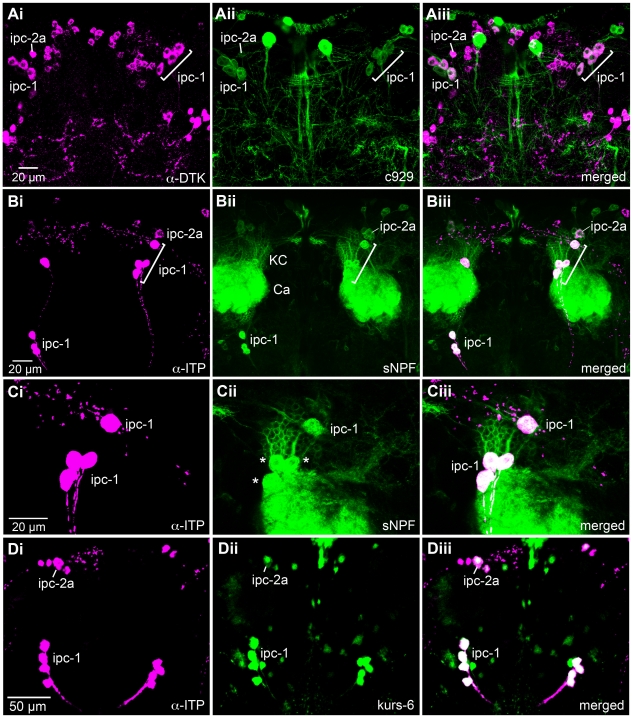
Co-expression of peptide and c929 or Kurs-6 Gal4 expression in neurosecretory cells of the adult brain. All images show brain in frontal view; Gal4-GFP expression is shown in green immunolabeling in magenta. **Ai - iii** Co-expression of DTK-immunolabeling and c929 expression in ipc-1 and 2a cells. These neurosecretory cells are the only ones coexpressing the two markers. **Bi - iii** Co-expression of ITP immunolabeling and *snpf*-GAL4 –driven GFP in ipc-1 and 2a cells. Note the variable location of ipc-1 cell bodies. The intrinsic Kenyon cells (KC) of the mushroom bodies express sNPF and their dendrites in the calyces (Ca) are seen here. **Ci - iii** The same section in higher magnification showing double labeled ipc-1 cell bodies in the right brain hemisphere. In **Cii** the ipc-1 cells are indicated by asterisks. **Di - iii** Co-expression of ITP-immunolabeling and Kurs-6-Gal4 expression in ipc-1 and 2a cells. Note that only one (the large ipc-2a) of the four ipc-2 cells coexpress the two markers.

Next, we asked whether the ipc-1 and ipc-2a neurons express any further peptides. Two candidate peptides emerged after testing a large number of peptide antisera: *Drosophila* tachykinin (DTK) and short neuropeptide F (sNPF). Thus, in adult brains with c929-Gal4 driven GFP, that were used for immunolabeling with antisera to DTK or sNPF, we detected both peptides in the ipc-1 and ipc-2a neurons (Fig. 3A – C, S1A - C), but in no other c929-expressing neurons. In addition, colocalization of ITP, DTK and sNPF in the ipc-1 and ipc2a neurons was confirmed by using sNPF-Gal4 driven GFP combined with immunolabeling with ITP or DTK antisera (Fig. 3B – C). The expression pattern of the sNPF-gal4 line used (NP6301) has previously been confirmed with antiserum to sNPF [24] and is shown here to include also the ipc-1 and ipc-2a neurons.

Furthermore, we show that another enhancer trap Gal4 line, Kurs-6 [9], is expressed in ipc-1 and ipc-2a neurons by using antiserum to ITP as a marker (Fig. 3D, S1D). When immunolabeling brains expressing Kurs-6-driven GFP with antiserum to DTK we found immunoreactivity only in the ipc-1 and ipc-2a cells of all the GFP labeled neurons. With all markers the cell bodies of the ipc-1 neurons (but not the ipc-2a) display variable locations along the axonal tracts of the lateral neurosecretory cells in the protocerebrum (Fig. S1D – F).

**Figure 4:**
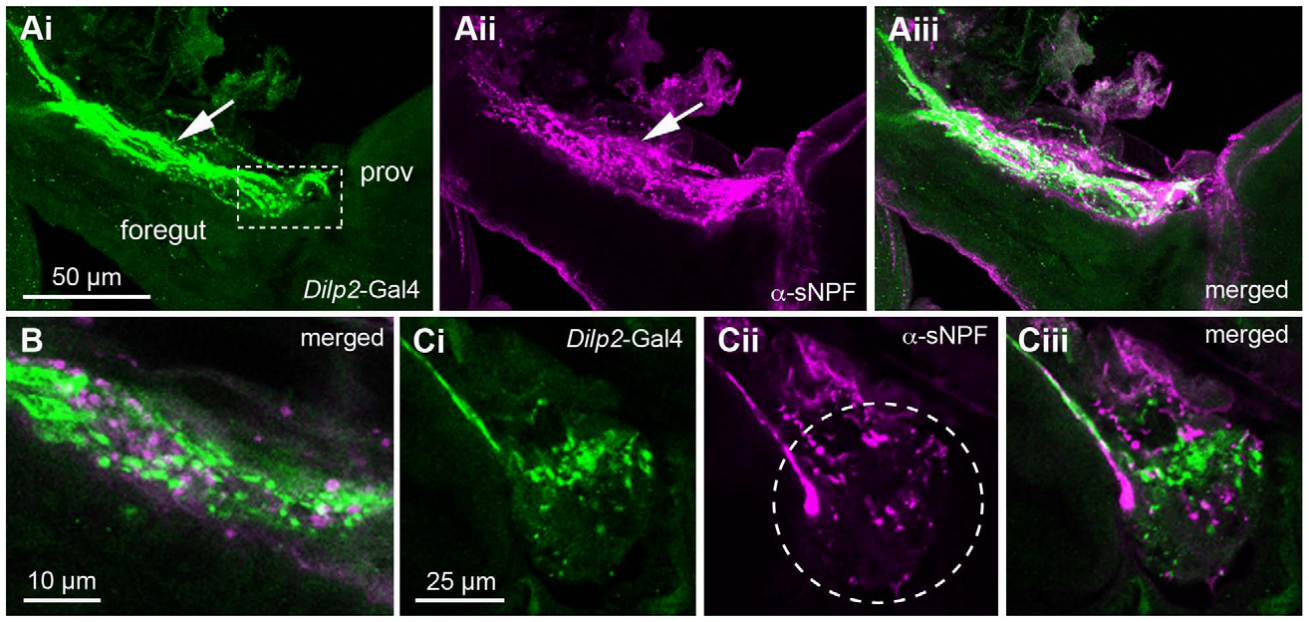
sNPF-immunolabeling in axons supplying the retrocerebral complex. Wholemount preparations of the foregut (or esophagus), proventriculus (prov) with attached corpora cardiaca-recurrent nerve and corpora cardiaca-hypocerebral ganglion (corpora allata not seen here). As a marker to outline the retrocerebral complex *Dilp2*-Gal4-driven GFP was used to reveal axons of insulin producing cells. It is clear that the DILP2 expressing median neurosecretory cells in the brain do not co-express sNPF or DTK (not shown). Thus, the occational “white profiles” in the merged images are caused by close superposition of two separate neurons. **Ai - iii** Foregut and proventriculus with the corpora cardiaca nerve (arrow) and corpora cardiaca with hypocerebral ganglion (boxed) with axons expressing *Dilp2* and sNPF-immunolabel. These panels show projections of several optic sections, thus some *Dilp2* and sNPF expressing axons superimpose in Aiii. **B** A higher magnification of the corpora cardiaca nerve separate varicose axons express *Dilp2* and sNPF (no colocalization). **Ci - iii** Detail of the corpora cardiaca region with the two markers in separate varicose axons.

The varicose axons of the ITP-immunoreactive ipc-1 and 2a neurons extend into the corpora cardiaca as well as the anterior aorta and intestine with crop duct [15]. We tested whether axon terminations in these areas also display DTK and sNPF immunoreactivity. Thus, we applied antisera to the two peptides on dissected brains with attached anterior intestines, corpora cardiaca, hypocerebral ganglion, and aorta. As a marker for these sites we employed flies expressing *dilp2*-Gal4 driven GFP, that visualizes neurosecretory cell axons expressing insulin-like peptide 2 (DILP2) that terminate in the same region. No ipc-2a (or other ipc-2) cells express ITP in the larva. [17], [47]. We found that both sNPF and DTK immunoreactive axons extend to the same areas as shown for ITP (Fig. 4). These axons run in close proximity to the DILP2-expressing ones, but as expected no colocalization was seen. This is also obvious from the immunolabeling in the brain where DILP2 expressing median neurosecretory cells do not co-express sNPF or DTK (not shown).

In summary, we have identified five pairs of large protocerebral neurosecretory cells, the ipc-1 and ipc-2a neurons, that co-express three neuropeptides ITP, DTK and sNPF in the brain of adult *Drosophila*. These cells are also included in the expression pattern of two Gal4-lines, c929 and Kurs-6. Both the sNPF and the DTK distribution superimpose with the c929 and Kurs-6 expression only in the ipc-1 and 2a neurons.

### Analysis of ITP, DTK and DIMM in larval brain neurosecretory cells

**Figure 5:**
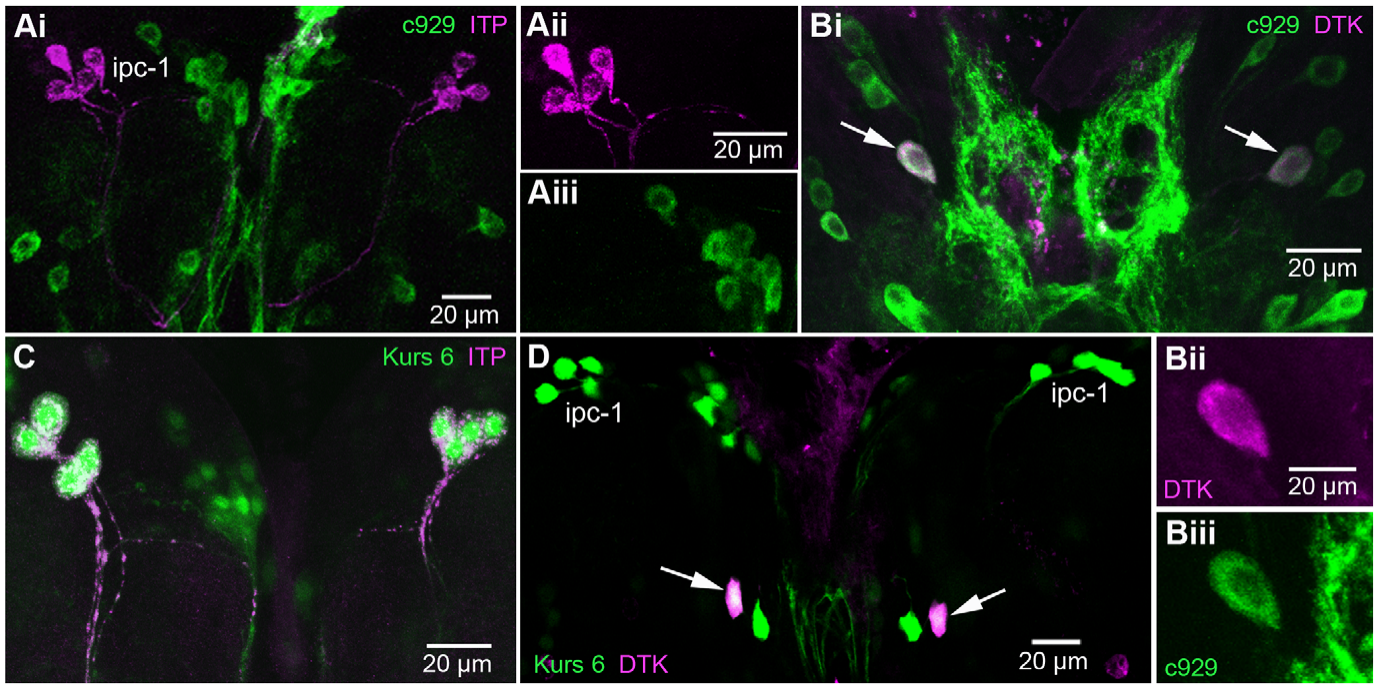
In the brain of the third instar larva the large neurosecretory cells do not coexpress marker. The brain of later third instar larva is shown in dorsal view, anterior is at top of images. The Gal4-GFP expression is shown in green, immunolabeling in magenta. **Ai – iii** The four pairs of ITP immunolabeled ipc-1 cells do not express c929 (single channels are shown in ii and iii). **Bi – iii** Only two neurons (arrows) coexpress DTK immunolabeling and c929 (single channels in ii and iii). These are descending interneurons with extensive axonal projections into the ventral nerve cord (not shown)[37]. **C** The ipc-1 neurons coexpress ITP immunolabel and Kurs 6 expression (this GFP is seen also in nuclei). **D** The four pairs of ipc-1 neurons express Kurs 6, but not DTK immunolabel. The large descending neurons (arrows), however, coexpress the two markers.

In the larval brain only the ipc-1 neurons express ITP, [15], but neither DTK nor sNPF could be detected in ipc-1-like cells in larvae [24], [37]. Here we tested whether the ipc-1 cells are included in the larval c929 expression pattern by immunolabeling with ITP antiserum. As seen in Fig. 5A, the ipc-1 cells express ITP, but not c929-driven GFP. However, the Kurs-6 driver is expressed in the ipc-1 cells (Fig. 5C). We also applied antiserum to DTK to larval brains expressing c929-driven GFP and found co-expression of the two markers only in a pair of large descending neurons (Fig. 5B), known from an earlier study [37]. Using the Kurs-6 driver to display GFP we did not see any colocalization with DTK in the ipc-1 cells, but again the markers were both seen in the large descending neurons (Fig. 5D). It was previously shown that sNPF is not detectable in any of the c929 expressing cells of the larva [24]. These findings taken together suggest that only in adult brains there is a detectable c929 (DIMM) expression in neurosecretory cells (ipc-1 and 2a) producing ITP, DTK and sNPF. Also, it is likely that using the c929 line to drive RNAi will primarily affect peptide levels in ipc-1 neurons in stages after the third instar larva. Similarly, the Kurs-6 driver should only affect ITP levels in the ipc-1 neurons of the larva, but not sNPF or DTK.

### Peptide knockdown affects sensitivity to desiccation

Previous studies in locust have shown a role of ITP in water and ion reabsorption [48]. This may suggest the possibility that the ipc-1 and 2a neurons in *Drosophila* utilize ITP and the colocalized peptides for hormonal control of anti-diuresis. There are also indications that tachykinin-related peptides, like DTKs, affect secretion in Malpighian (renal) tubules is some insects [49], [50] and at least one of the two DTK receptors is expressed in renal tubules, crop and intestine of *Drosophila* [51], [52]; see also http://www.flyatlas.org/ [53]. These findings prompted us to investigate whether the peptides in the ipc-type neurosecretory cells play a role in responses to desiccation.

To address this, we employed targeted RNAi to specifically knock down the expression of DTK and sNPF in the ipc-1 and ipc-2a cells using two different Gal4 drivers, c929 and Kurs 6, crossed to either of the transgenes UAS-*dtk*-RNAi or UAS-*snpf*-RNAi. Unfortunately, the cross between c929-Gal4 and UAS-*itp*-RNAi (from VDRC, Vienna) transgenes was found to be lethal at the early larval stages (H. Dircksen, unpublished observation) and therefore ITP knockdown was not studied here. The flies with sNPF or DTK levels knocked down were monitored for the effect of desiccation on life span and water retention. Only male flies were used for all experiments. As controls we employed the parental Gal4 and UAS strains crossed with w^1118^ flies.

The two RNAi lines (*snpf* and *dtk*) used here have been extensively tested for their efficacy in diminishing RNA and peptide levels after global knockdown [36], [42], [43]. Here we employed immunocytochemistry to test the effect of targeted sNPF and DTK knockdown in the ipc-1 cells by the Kurs6 Gal4 driver. As seen in Fig. S2 the immunolabeling of the ipc-1 neurons was diminished by almost 70% for sNPF and more than 50% for DTK compared to ipc-1 cell bodies in control flies.

**Figure 6:**
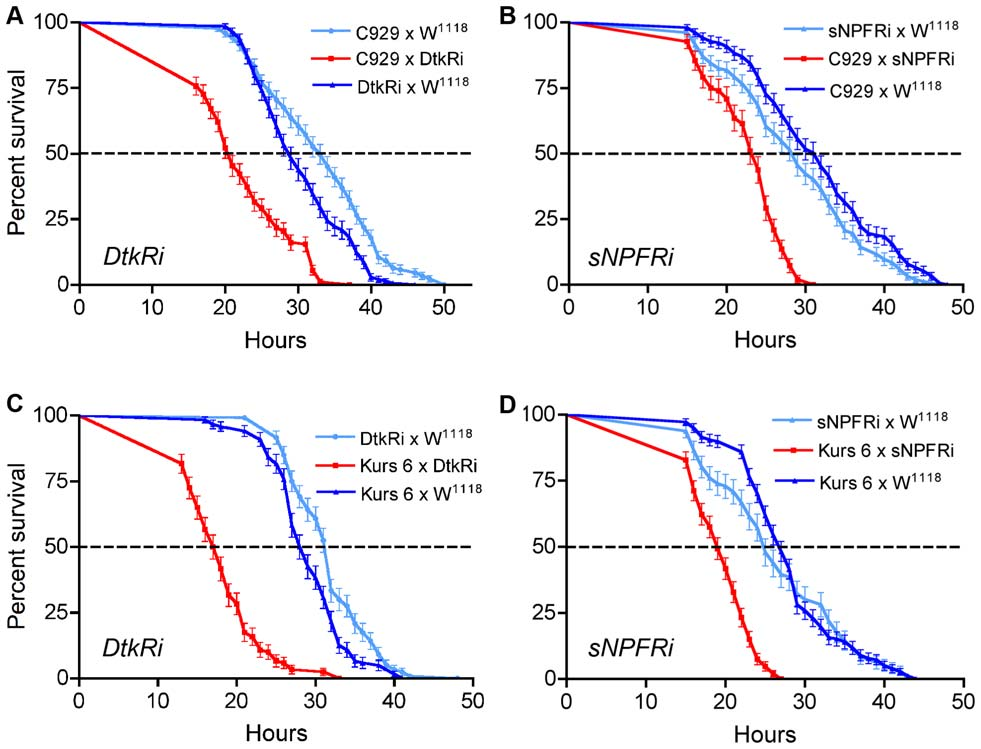
Knockdown of sNPF and DTK in neurosecretory cells increases sensitivity to desiccation. Two different Gal4 drivers that specify the ipc-1 and 2a cells were used to knock down levels of DTK or sNPF in these cells. Flies were kept singly in tubes with neither food nor water and each hour (starting after 12 h) dead flies were counted. Each of the four knockdown experiments (red curves) displayed a significant decrease in survival at desiccation. As controls the two parental strains crossed to w^1118^ flies were used. All experiments were run in triplicate (three separate fly crosses per genotype) with a minimum of 40 flies of each genotype in each replicate (except for one control in B and D) The statistics used for trends in survival was a Log-rank test (Mantel-Cox). The survival curves are displayed as averages of the three replicates with standard errors indicated by bars. **A** Knockdown of DTK in ipc-1 and 2a cells with the c929 enhancer trap Gal4 line (C929) crossed with UAS-*Dtk*-RNAi (DtkRi). The survival curve of knockdown flies was significantly different from that of the two controls (P<0.001, Log-rank test; for each genotype n = 139–171). **B** Flies bearing the transgenes c929-Gal4 and UAS-*snpf*-RNAi (sNPFRi) displayed a similar decrease in survival at desiccation [P<0.001, Log-rank test; for each genotype n = 153, 154, 96 (sNPFRi x W^1118^)]. **C** The Kurs 6 Gal4 driver produced a drastic phenotype when crossed to UAS-*Dtk*-RNAi flies. A dramatic decrease in survival was observed: median lifespan of peptide knockdown flies was reduced by 10 h compared to the controls (P<0.001, Log-rank test; for each genotype n = 120). **D** The flies from the cross between Kurs 6 and UAS-snpf-RNAi displayed a drastic reduction in survival [P<0.001, Log-rank test; for each genotype n = 146, 178 and 96 (sNPFRi x W^1118^)].

Flies exposed to desiccation (without access to food or water) were kept individually in tubes under controlled conditions and their survival was monitored. The flies with diminished DTK level in the ipc-1 and 2a neurons using the c929 driver displayed shorter life spans compared to their parental controls at desiccation (Fig. 6A). The median life span was 21 h for the DTK-knockdown flies and for controls 29 h and 33 h, respectively, i. e. about 30% reduction [P<0.001 compared to each control, Logrank test (Mantel Cox); n = 140–170 for each genotype]. The desiccated Kurs6/*dtk*-RNAi flies displayed an even more drastically abbreviated survival time compared to controls (Fig. 6C). Here median life span of DTK knockdown flies was 17.5 h, whereas those of controls were 28.5 and 32 h, which is a reduction of 38–45% (P<0.001 compared to each control, Logrank test; n = 120 for each genotype). Next we showed that knockdown of sNPF by c929-Gal4 (c929/*snpf*-RNAi) and Kurs-6-Gal4 (Kurs6/*snpf*-RNAi) also resulted in a significant decrease in survival time of desiccated flies (Fig. 6B, D). With the c929 driver the sNPF knockdown resulted in a median survival of 23 h compared to controls at 29 and 31 h equaling an approximate 21–26% decrease (P<0.001 compared to each control, Logrank test; n = 96–154 for each genotype). With the Kurs 6 driver the median survival is 19 h for sNPF knock-down and 25 h and 27 h for controls, which is a 24–30% reduction (P<0.001 compared to each control, Logrank test; n = 96–178 for each genotype). In summary, knockdown of DTK or sNPF with either of the two Gal4 drivers led to a highly significant decrease in life span at desiccation.

**Figure 7:**
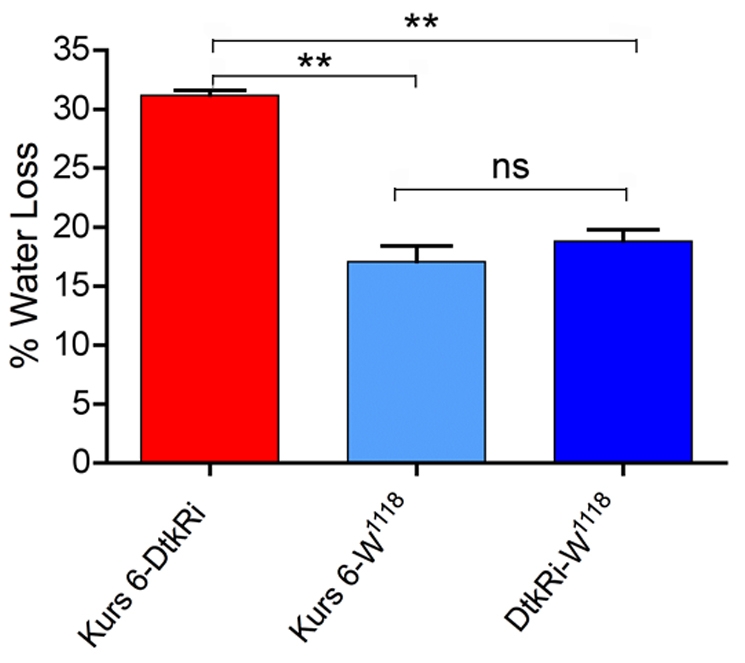
Water loss increases in peptide-knockdown flies exposed to desiccation. Flies with DTK diminished with the Kurs 6 Gal4 driver were exposed to desiccation (no food and no water) for 16 h. The whole body water content was calculated in the desiccated flies and in flies of the same genotypes that were normally fed (see Material and methods). The water loss is given in the graph for the experimental and control flies. Each genotype was tested in three replicates. A significant increase in water loss was seen in the flies with diminished DTK in ipc-1 and 2a cells (One-way ANOVA, with Bonferroni's multiple comparison, P<0. 001; for each genotype n = 100–170).

Since desiccated flies with diminished levels of peptides displayed reduced survival compared to controls we hypothesized that they lost more water. Thus, we measured body water in flies exposed to 16 h desiccation (no food or water) compared to flies kept with normal access to food and water. Water loss over 16 h was determined for each genotype by subtracting the calculated water content at 16 h from that at 0 h. Since dead dry weight was necessary to obtain we had to use separate flies for 0 h and 16 h. We tested the fly cross that displayed the most drastic reduction in survival at desiccation: Kurs 6 driven DTK knockdown. As seen in Fig. 7, DTK knockdown in ipc-1 and 2a cells led to a significant increase (nearly a doubling) in water loss at desiccation compared to controls (Oneway ANOVA, Bonferroni's test P<0. 001; for each genotype n = 100–170).

### Peptide knockdown also affects sensitivity to starvation

In locusts tachykinin-related peptides have been shown to be upregulated in the intestine at nutritional stress, such as starvation [54], [55], and sNPF in *Drosophila* has been associated with feeding and regulation of growth [42], [43]. Thus, we were interested to see whether knockdown of DTK and sNPF in the ipc-1 and 2a cells would affect responses to starvation. To test this we put transgenic flies under nutritional stress (access to water, but not nutrients) and monitored their survival.

**Figure 8:**
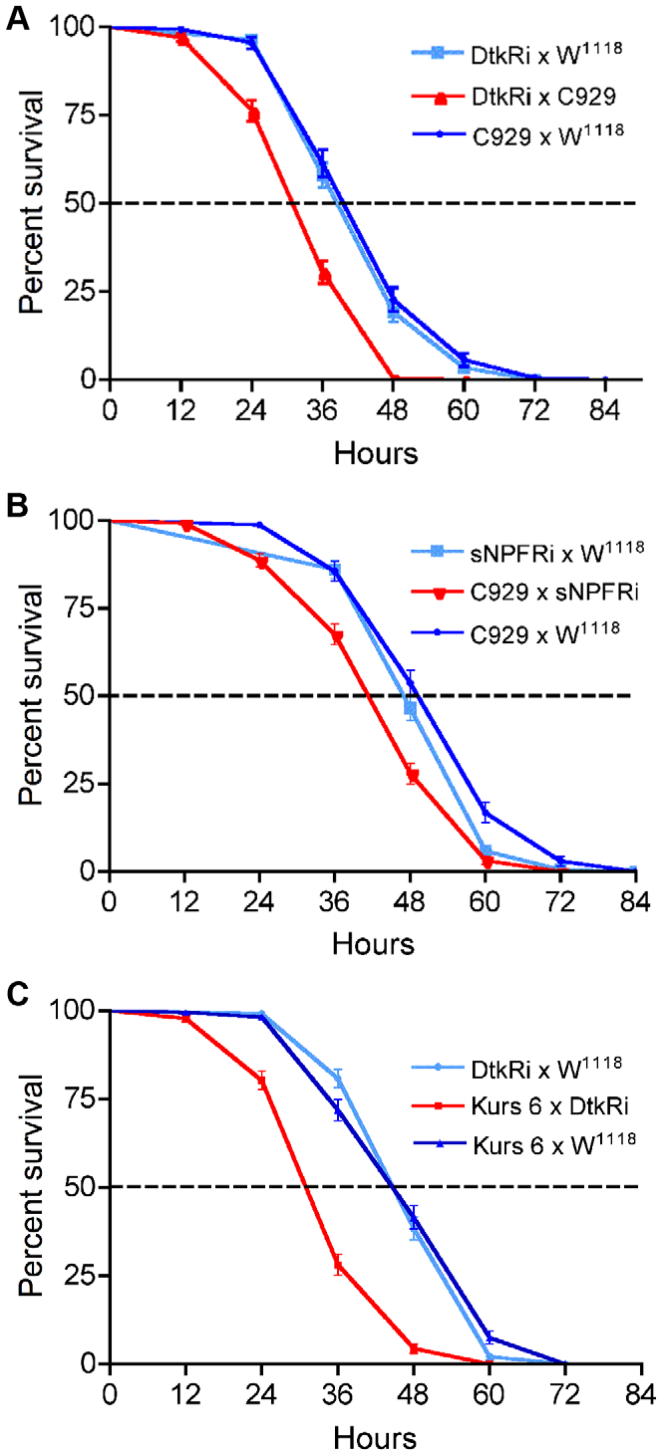
Sensitivity to starvation increases after knockdown of sNPF and DTK in neurosecretory cells. The two Gal4 drivers, c929 and Kurs 6 were used to specify knock down levels of DTK or sNPF in the ipc-1 and 2a cells. Flies were kept individually in tubes supplied with aqueous agarose, but no food. Dead flies were monitored every 12 h and experiments were run in triplicates (n =  at least 46 flies per genotype and replicate), otherwise the experimental procedure and statistics were as in Fig. 6. In each of the experiments the peptide knockdown resulted in flies with significantly diminished survival at starvation. **A** Knockdown of DTK in ipc-1 and 2 cells with the c929 driver (P<0.001, Log-rank test; for each genotype n = 139–171). **B** Knockdown of sNPF with c929 driver (P<0.001, Log-rank test; for each genotype n = 166–247). **C** Kurs 6-driven knockdown of DTK produces strongly diminished survival at starvation (about 12 h decrease at 50% survival) (P<0.001, Log-rank test; for each genotype n = 224–229).

We found that flies with down-regulated DTK in the ipc-1 and ipc-2a cells (c929/*dtk*-RNAi) displayed shorter life span compared to parental controls when starved (Fig. 8A). Median life span was decreased by about 23% from 39 to 30 h (P<0.001 compared to each control, Logrank test; n = 140–170 for each genotype). Similarly, knocking down sNPF in the same sets of neurosecretory cells led to flies with abbreviated life span at starvation (Fig. 8B), although less pronounced at about 15% (P<0.001 compared to each control, Logrank test; n = 166–247 for each genotype). Again, we employed the other Gal4 line, Kurs-6-Gal4, to drive RNAi. Indeed, DTK knock-down with the Kurs-6 driver also led to a significant decrease in survival time of the starved flies (Fig. 8C). The reduction in median life span is about 25% (P<0.001 compared to each control, Logrank test; n = 224–229 for each genotype).

In conclusion, reducing DTK or sNPF levels in the ipc-1 and ipc-2a cells both lead to increased sensitivity (or decreased resistance) to both starvation and desiccation.

### Effects of peptide knockdown on locomotor activity at starvation

**Figure 9:**
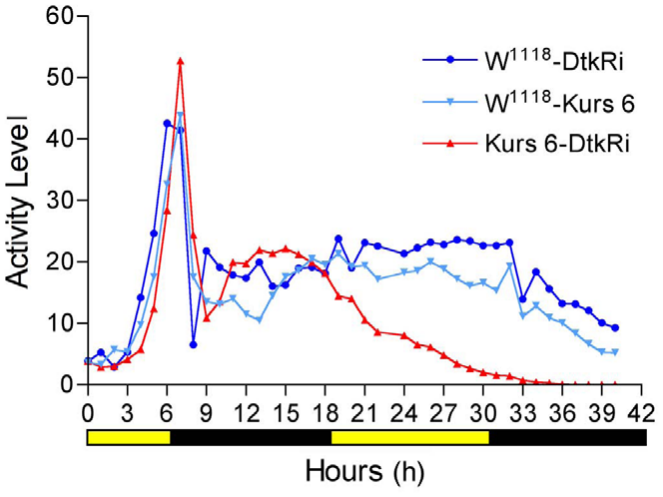
Locomotor activity in transgenic flies at starvation. Flies were tested for locomotor activity during starvation. DTK knockdown flies generated by the cross of Kurs 6-Gal4 and UAS-*Dtk*-RNAi flies (Kurs 6-DtkRi) and parental controls (w^1118^-Kurs 6 and w^1118^-DtkRi) were kept individually in glass tubes with aqueous agarose in one end, under 12∶12 light:dark (LD) conditions at 25°C, and their locomotor activity was recorded in a Trikinetics activity monitor system. We display average activity (arbitrary units) of all flies of each genotype over 40 h, starting 3 h after onset of starvation. In the graph we set the time 0 h at onset of recording (which started 5.5 h after lights on). The LD phases are indicated by yellow/black bar below. Flies of all three genotypes displayed the same locomotor activity patterns until 18 h of recording. At the end of the light phase an increased activity was seen, corresponding to evening activity (evening anticipation). This declines over about 2 h into the dark phase where a trough level is reached at about 8 h of monitoring. The flies thereafter increase their activity to a steady intermediate level over the remaining recording. This increased activity during the dark phase is likely to reflect food search activity. At 18 h the peptide knockdown flies start to die, but continued recording shows that control flies continue the same level of activity and no morning or evening peak of activity can be seen on day two. The experiment was made in two replicates with a total of 56–81 flies of each genotype still alive at 18 h of recording.

Flies that are deprived of food increase their locomotor activity after about 12 h, probably indicating that they are searching for food [26], [27]. The same two papers also showed that deletion of cells producing the peptide hormone AKH led to strongly decreased locomotor activity at starvation. To test whether knockdown of DTK in ipc-1 and 2a cells affect food search behavior we monitored locomotor activity of the transgenic flies at starvation. Flies were kept individually in tubes supplied with aqueous agarose for 40 h in a Trikinetics activity monitor under a 12∶12 L:D cycle. Activity recordings were started in late light phase (about noon). We chose the Kurs 6-driven knockdown of DTK for tests, since these flies displayed the strongest decrease in survival at starvation (see Fig. 8C). During starvation both the Kurs6/*dtk*-RNAi flies and the two controls displayed normal locomotor activity for about 12 h after onset of food deprivation (Fig. 9). This includes the evening activity before and after lights off. After a brief return to low activity, characteristic for the dark phase, their activity increased slightly and continued steadily at an intermediate level throughout the dark phase. Thus, over the 40 h recording neither of the genotypes displayed any L:D-related fluctuations of activity after 12 h of starvation (Fig. 9). Flies with DTK levels knocked down started to perish after about 18 h of recording. Thus, the average activity of the genotypes is best compared between 0–18 h (i. e. until 21 h starvation; see legend Fig. 9). Over this period we detected no difference between flies with DTK diminished in ipc neurons and the controls. Thus, locomotor activity does not seem to be influenced by loss of ipc-1 and 2a signaling.

## Discussion

We describe the colocalization of peptides derived from three different precursor genes in a small set of large neurosecretory cells in the adult *Drosophila* brain. These cells, designated ipc-1 and 2a, express ITP, DTKs and sNPFs and are part of neuron populations defined by the enhancer trap Gal4 lines c929 and Kurs-6 (see Fig. 1). This enabled us to diminish the levels of sNPF and DTK specifically in the two cell groups in adult flies by targeted RNAi using the two Gal4 drivers and monitor the effect on stress resistance, water retention and locomotor activity. Both Gal4 driver lines crossed with either of the UAS-*dtk*-RNAi or UAS-*snpf*-RNAi flies produced the same phenotypes with minor differences in strength. Flies with diminished peptide levels in ipc-1 and 2a cells displayed increased sensitivity to stress in the form of desiccation and starvation, as monitored by their survival, and also a decrease in water retention at desiccation. The locomotor activity of DTK-knockdown flies at starvation was not affected compared to control flies suggesting that food search behavior was not affected by loss of DTK signaling.

It is likely that the hormonal roles of brain-derived DTKs and sNPFs are specific to adults flies, based on the following arguments. In a previous study it was shown that only the four pairs of ipc-1 cells display ITP expression in the larval brain, and the ipc-2 cluster (including ipc-2a) appear during late pupal development [15]. More important is the finding that the DTK and sNPF immunolabeling of the ipc-1 (and ipc-2a) cells can be seen only in the adult flies. Furthermore, we have shown that in the larva coexpression of DTK and DIMM, as revealed by c929, is detected in only two large brain interneurons and not in neurosecretory cells (see also [46]) and there is no coexpression at all of sNPF and c929 in the CNS of larvae [24]. Surprisingly, we could not detect c929 expression in the ITP immunolabeled larval ipc-1 neurons, although they are likely neurosecretory cells and these commonly express DIMM [45], [46]. Together this suggests that the four pairs of large ipc-1 and one pair of ipc-2a neurons start expressing sNPF, DTK and DIMM (at least c929) in the adult flies (or during pupal development). In fact, in a previous study [37] DTK could not be detected in any brain neurosecretory cells of recently hatched adults, and our findings here therefore suggest that the DTKs in these cells are expressed in detectable amounts only in flies that are at least one day old. Consequently, it may be that both sNPF and DTK are recruited as brain-derived peptide hormones in adult flies when the ipc-1 and 2a cells start expressing the peptides and DIMM and thus the function of the two peptides as circulating hormones may be specific to the mature fly.

Hormonal roles of sNPF and DTK have not been clearly determined in any insect. Both peptides are known to display stimulatory activity on intestinal muscle in different insect species [34], [40], [56]. In addition, sNPF is implicated in feeding and growth in *Drosophila* [42], [43] and food search in mosquitoes [57] and tachykinins display weak diuretic action on renal tubules of moth and locust [49], [50]. Our findings here suggest that the peptides link to feeding and metabolism, as well as to stress. Loss of either of the two peptides in the ipc-1 and 2a neurons also diminished the flies' water conservation at desiccation. Thus the peptides of these neurons may play direct or indirect roles in anti-diuresis. A role in water and ion control would not be surprising since the colocalized peptide ITP is likely to be anti-diuretic [15], [35], [48]. Possibly ITP and the other peptides are released when the insect has depleted energy reserves and water to avoid further desiccation. Thus at starvation and desiccation the ipc-1 and 2a cells may be activated and the two peptides analyzed here, sNPFs and DTKs, may be accessory to the hormonal ITP. It might be that they act on different target organs/cells to orchestrate a response to the stressful situation.

We cannot exclude that sNPF and DTK released from the ipc-1 and 2a neurons target other brain neurosecretory cells that extend axons to the corpora cardiaca region. Two other peptide hormones released from the corpora cardiaca of *Drosophila*, DILPs and AKH, have clear roles in regulation of circulating carbohydrate levels and in responses to starvation and stress [2], [3], [26], [27], [47], [58], [59]. Our data do not support stimulatory action of ipc-derived peptides on DILP production or release for the following reasons. Previous work has indicated that sNPF derived from other neurons can stimulate production of DILPs in median neurosecretory cells of the *Drosophila* brain [43], [60]. The findings in our study suggest, however, that knockdown of sNPF and DTK in ipc neurons produces a phenotype opposite to what would be expected if these peptides stimulate insulin signaling [58], i. e. we observe increased sensitivity to stress. We cannot totally exclude a role of sNPF and DTKs in regulation of AKH levels, since *in vitro* work on locust corpora cardiaca has shown that tachykinins induce release of AKH [61]. On the other hand, our finding that locomotor activity levels at starvation (presumed food search behavior) are the same in DTK knockdown and control flies suggest that DTK does not directly affect AKH levels. Earlier work has namely shown that deletion of AKH producing cells abolishes starvation-induced increase of locomotor activity [26], [27].

Since all three peptides of the ipc-1 cells can be detected in axons extending to the posterior esophagus and the crop duct a direct action on this part of the intestine is possible. A similar innervation of the anterior gut has been seen by axons from lateral neurosecretory cells that express corazonin [31] and it has been suggested that corazonin may have a role in nutritional stress as well as in regulating the crop duct and thus release of nutrition into the intestine [62].

Our study has identified three neuropeptides in a distinct set of brain neurosecretory cells with axon terminations in neurohemal organs that may have pleiotropic roles as circulating hormones. These roles include homeostatic regulation at starvation and desiccation. Clearly it would be desirable to identify the exact targets of these peptides. One means to do this would be by identifying the expression sites of the receptors of ITP, sNPF and DTK. The ITP receptor has not yet been identified, and although the sNPF and DTK receptors have been identified and then characterized in different expression systems [52], [63], [64], [65], [66], their distribution outside the CNS has yet not been revealed in any detail. Also, it would be interesting to approach the functional role of ITP in nutritional stress responses.

## Materials and Methods

### Fly strains

Adult white-eyed flies *Drosophila melanogaster* (w^1118^ strain) as well as three lines of transgenic flies were used for immunocytochemistry and experiments. For some experiments late third instar larvae were utilized. The following Gal4 lines were used to drive the expression of green fluorescent protein (GFP) and for crosses to induce RNA interference (RNAi): c929-Gal4 (gift from P.H Taghert, St Louis, MO;[45]), Kurs-6-Gal4 (gift from G. Korge, Berlin, Germany; [9]), *Dilp2*-Gal4 (gift from Ping Shen, Athens, GA; [67]) and *snpf*-Gal4 (NP6301), from *Drosophila* Genetic Resource Center (DGRC), Kyoto Institute of Technology. Kyoto, Japan (see [24]). UAS-*mcd8-gfp* or UAS-*s65t-gfp* flies, from Bloomington *Drosophila* Stock Center (Univ. Indiana, Bloomington, IN) were used to visualize Gal4 expression.

For RNAi experiments we used the following strains: UAS-*dtk*-RNAi37A; UAS-*dtk*-RNAi 37D [36] and UAS-*snpf*-RNAi;;UAS-*snpf*-RNAi (the single insertion flies a gift from K. Yu, Daejeon, Korea; [42]) to knock down levels of DTK and sNPF, respectively. The efficacy of both these RNAi constructs, both at RNA and protein levels, has been reported [36], [42]. All flies were kept at 25°C on a 12∶12 h light/dark cycle and maintained on a diet of standard *Drosophila* medium.

### Antisera and immunocytochemistry

For immunocytochemistry adult *Drosophila* heads or nervous systems of third instar larvae were dissected in 0.01 M phosphate-buffered saline with 0.5% Triton X-100, pH 7.2 (PBS-Tx) and fixed in ice-cold 4% paraformaldehyde in 0.1 M sodium phosphate buffer pH 7.4 (PB) for 4 hours. Following rinsing with 0.1 M PB adult brains or larval CNS were either dissected out for whole mount immunocytochemistry or whole heads were incubated overnight in 20% sucrose in 0.1 M PB at 4°C as cryoprotection. Cryostat sections (50 µm thick) of the heads were cut on a cryostat at −23°C.

Incubation with primary antiserum for whole mount tissues was performed for 72 h, while sections were incubated overnight, both at 4°C. The following primary antisera were used: a rabbit antiserum to a generic sequence of insect tachykinin-related peptides (anti-LemTRP-1, code K-9836; [55]), known to recognize *Drosophila* DTKs [37]; at a dilution of 1∶2,000, a rabbit antiserum to a sequence of the *Drosophila* short neuropeptide F precursor (anti-sNPFp) [44] used at a dilution of 1∶4,000, and a rabbit antiserum raised against a sequence of locust ion transport peptide (ScgITP), known to recognize *Drosophila* ITPs [15], [68] used at a dilution of 1∶1,500.

For detection of primary antisera Cy3-tagged goat anti-rabbit antiserum (Jackson Immuno Research) was used at a dilution of 1∶1,000. Tissues or sections were rinsed thoroughly with PBS-Tx, followed by a final wash in PBS and then mounted in 80% glycerol in PBS. For each experiment at least 10 adult brains and 5 larval CNS were analyzed.

### Image analysis

Specimens were imaged with Zeiss LSM 510 META confocal microscope (Jena, Germany) using 20× or 40× oil immersion objectives. Confocal images were obtained at an optical section thickness of 0.2–0.5 µm and were processed with Zeiss LSM software. Images were edited for contrast and brightness in Adobe Photoshop CS3 Extended version 10.0.

### Quantification of immunofluorescence after targeted RNAi

To determine levels of sNPF and DTK after RNAi we applied immunocytochemistry as above. Dissected 5–6 day old male brains were used in experiments. Specimens were imaged under identical conditions. Immunofluorescence was quantified in single optical sections in set regions of interest (ROI) using ImageJ v1.42, NIH, Bethesda, ML (http://rsb.info.nih.gov/ij). Fluorescence was quantified in several adjacent ROI to cover the entire cell bodies of ipc-1 and control neurons. To account for possible differences in immunofluorescence between different specimens, i. e. differences not produced by RNAi, a group neuronal cell bodies (LPP and LNC) located adjacent to the neurons of interest (ipc-1) were utilized as internal controls. These control neurons were not included in the Kurs 6- or c929-Gal4 expressions and thus not targeted by RNAi. Five brains per genotype were measured. The data were analyzed using Prism v4.0 (GraphPad, CA).

### Assays of survival during starvation and desiccation

Starvation experiments were performed according to the protocol of Lee and Park [27]. For peptide knockdown we used two Gal4 drivers (c929 and Kurs 6) to drive RNAi (UAS-*snpf*-RNAi and UAS-*Dtk*-RNAi) specifically in ipc-1 and 2a cells. As controls we used the parental strains crossed to w^1118^. For starvation male flies, aged 4–6 days, were anesthetized on ice and then placed individually in 2 ml cotton-capped glass vials containing 500 µl of 0.5% aqueous agarose. All vials were placed in an incubator with 12∶12 light:dark conditions at 25°C. The vials were checked for dead flies every 12 hours until no living flies were left. For desiccation experiments same protocol was followed, except that the vials were empty (no food or water) and the vials were checked hourly until there were no living flies. All experiments were run in triplicate with at least 40 flies of each genotype in each run.

### Measurement of water loss

Male flies were exposed to 16 hours desiccation to determine water loss (testing peptide knockdown and parental strains). Two groups of flies of each genotype were weighed: (1) normally fed flies (0 hour desiccation) and (2) after 16 hours desiccation with no food and no water (16 hours desiccation). To obtain water content groups of 5 male flies were weighed (Sartorius, Göttingen) after anesthetizing them on ice (living wet weight) and were subsequently dried at 60°C for 24 hours. Dry flies were weighed after reaching room temperature (dry weight). Water content was calculated by subtracting the dry weight from the wet weight. Water loss over 16 h was calculated for each genotype by subtracting the water content at 16 h from that at 0 h. Since dead dry weight was necessary to obtain we had to use separate flies for 0 h and 16 h. Experiments were run in triplicate with at least 33 flies of each genotype and replicate (n = 100–170 for the three genotypes).

### Recording of locomotor activity

The locomotor activity of different genotypes was analyzed with a Trikinetics activity monitor (Trikinetics, Brandeis CA, USA). Single flies were placed in monitoring glass tubes (5 mm diameter) filled in one end with 2 cm of 0.5% aqueous agarose. Tubes were placed in Trikinetics monitoring racks in an incubator at 25°C with a light-dark cycle of 12∶12 h. All monitoring of activity started 3 h after onset of starvation (recordings started at 5.5 h after lights on) and activity data (crossings of an infrared beam) were collected by the Trikinetics computer software in bins every 15 minutes. Data were collected for 40 h and activity records from 79–81 individual flies of each genotype (in two replicates) were pooled to obtain average activity levels. The data were analyzed using Microsoft Excel.

### Data analysis

Data was collected and analyzed in Microsoft Excel and statistical analysis was performed with Prism GraphPad v5.0.2. For survival curves obtained in starvation and desiccation assays, log-rank (Mantel-Cox) test was performed to analyze the trends in lifespan. For water retention assays a one-way ANOVAs was used to compare the water loss levels in desiccated flies.

## Supporting Information

Figure S1 Patterns of colocalized markers in peptidergic ipc-1 and 1c-2a cells. Frontal views of the adult brains showing Gal4-driven GFP (green) and peptide immunolabeling (magenta). Ai - iii DTK-immunolabeling and snpf-Gal4 expression in ipc-1 cells in both hemispheres of the brain. Bi - iii Co-expression of ITP-immunolabeling and snpf-GAL4 expression in ipc-1 cells (arrows in Bii). Also the intrinsic neurons of the mushroom bodies express sNPF-GFP and their densely packed dendrites in the calyces (Ca) are visible. Ci - iii Co-expression of sNPF immunolabeling and c929 expression in ipc-1 cells (arrow) and ipc-2a. D Co-expression of ITP-immunolabeling and Kurs-6-GFP in ipc-1 (arrows) and ipc-2a cells. E and F Variability in location of cell bodies of ipc-1 neurons labeled with ITP antiserum and c929-GFP. In spite of the variable cell body locations the axons join the same tract (arrows) to the corpora cardiac nerves.
(6.87 MB TIF)

Figure S2 Targeted RNAi diminished sNPF and DTK immunolabeling in ipc-1 neurons. We used the Kurs6-Gal4 to drive snpf- and dtk-RNAi and monitored levels of the peptides in ipc-1 neurons by immunocytochemistry. A1–4 Kurs6-driven snpf-RNAi diminishes sNPF imunolabeling selectively in ipc-1 neurons (asterisks). We utilized Kurs6-driven GFP to clearly identify the ipc-1 cells at RNAi knockdown (A1–2). The dorsal cells (arrows) were used as internal controls (LNCs in Fig. S2C below). A4 is a single 1 µm optical section showing two of the ipc1-cells. B1–2 Immunolabeled ipc-1 neurons in a control brain (w1118/UAS-snpf-RNAi). Asterisks indicate the sNPF immunolabeled ipc-1 neurons. B2 shows cells in single optical section. C Quantification of relative fluorescence after Kurs6-driven snpf-RNAi. Immunofluorescence was measured in multiple cell bodies of ipc-1 and control (LNC; arrow in A3) neurons in 5 specimens of each genotype. A significant reduction of fluorescence was seen in the ipc-1 neurons compared to controls, sNPF-RNAi/+ (P<0.001; ANOVA). D1–3 Kurs6-driven dtk-RNAi diminishes DTK immunolabeling selectively in ipc-1 neurons (asterisks). Control neurons at arrow (LPP in Fig. S2F). D3 is a single 1 µm optical section of ipc-1 neuron. E Three DTK immunolabeled ipc-1 neurons in control brain (w1118/UAS-dtk-RNAi) shown in single optical section. F Quantification of relative fluorescence after Kurs6-driven dtk-RNAi. Immunofluorescence was measured in multiple cell bodies of ipc-1 and control (LPP; arrow in D2) neurons in 5 specimens of each genotype. A significant reduction of fluorescence was seen in the ipc-1 neurons compared to controls, dtk-RNAi/+ (P<0.01; ANOVA).
(7.42 MB TIF)
